# 

**Published:** 1889-12

**Authors:** 


### TABLE OF CONTENTS.

ORIGINAL AND SELECTED.

Clinical remarks on dyspepsia, by R. C. Word M. D. ... 441
Catarrhal Neuralgia, by A. G. Hobbs, M. D. ... 449
Grain of corn in the windpipe, by E. W. Roe, M. D. ... 453
Prevention of typhoid fever ... 455

ABSTRACTS &c.

Nutmeg poisoning; Treat. of warts; Chronic ulcers of the leg; Exalgine; Styrone as an antiseptic ... 457
Conjugal diabetes; Blood-tax of India ... 458
Whooping cough spasm ... 459
Cure of amenorrhœa; Death or abortion ... 460
Powder stains on the skin; Tongue-tie; Ichthyol in erysipelas; Veratrum Viride in pneumonia ... 461
Salol and sulphur; Washing out the abdominal cavity in peritonitis ... 462
Goitre treated with strophanthus; A novel tobacco antidote ... 463
Sulfonal for night-sweats ... 464
Acute intussusception in a child cured by operation ... 465
Salol and bismuth in diarrhœa; Cardiac displacement ... 466
Salix nigra; The new tenicides; Morphine, its excretion, &c ... 467
Acute articular rheumatism; Perosmic acid in epilepsy ... 468
Croup and diphtheria; Diphtheria; Homeriana in tuberculosis; Epistaxis ... 469

SCIENTIFIC.

Mixing of vegetables; Singing beach; Secondary chromatic aberration; Three new asteroids; Electric ... 470
Ventilating bees; Alga upon the hair; To preserve flowers; Protect plants from frost ... 471

NOTES AND FORMULÆ.

Cholera infantum; Chronic rheumatism; Thiol 472
Gastralgia; Nasal catarrh; Goitre; Treatment of asthma ... 473

EDITORIAL.

Valedictory ... 474
The true and the false ... 475
Mr. E. Amis; Book notices ... 476
Book notices ... 477
Medical News & Visiting List; Buffalo Lithia Springs; Thomas W. Hood; Henry R. Wampole & Co.; So. Med. Col. ... 478
Special notes ... 478, 479, 480

